# What Is life? Rethinking Biology in Light of Fundamental Parameters

**DOI:** 10.3390/life14030280

**Published:** 2024-02-20

**Authors:** Jacques Fantini, Mélanie Matveeva, Marine Lefebvre, Henri Chahinian

**Affiliations:** Department of Biology, Faculty of Medicine, University of Aix-Marseille, INSERM UMR_S 1072, 13015 Marseille, France; melanie.matveeva@etu.univ-amu.fr (M.M.); marine.lefebvre.1@etu.univ-amu.fr (M.L.); henrichahinian@gmail.com (H.C.)

**Keywords:** biology, origin of life, quantum biology, water, time, electrostatic potential

## Abstract

Defining life is an arduous task that has puzzled philosophers and scientists for centuries. Yet biology suffers from a lack of clear definition, putting biologists in a paradoxical situation where one can describe at the atomic level complex objects that remain globally poorly defined. One could assume that such descriptions make it possible to perfectly characterize living systems. However, many cases of misinterpretation put this assumption into perspective. In this article, we focus on critical parameters such as time, water, entropy, space, quantum properties, and electrostatic potential to redefine the nature of living matter, with special emphasis on biological coding. Where does the DNA double helix come from, why cannot the reproduction of living organisms occur without mutations, what are the limitations of the genetic code, and why do not all proteins have a stable three-dimensional structure? There are so many questions that cannot be resolved without considering the aforementioned parameters. Indeed, (i) time and space constrain many biological mechanisms and impose drastic solutions on living beings (enzymes, transporters); (ii) water controls the fidelity of DNA replication and the structure/disorder balance of proteins; (iii) entropy is the driving force of many enzymatic reactions and molecular interactions; (iv) quantum mechanisms explain why a molecule as simple as hydrocyanic acid (HCN) foreshadows the helical structure of DNA, how DNA is stabilized, why mutations occur, and how the Earth magnetic field can influence the migration of birds; (v) electrostatic potential controls epigenetic mechanisms, lipid raft functions, and virus infections. We consider that raising awareness of these basic parameters is critical for better understanding what life is, and how it handles order and chaos through a combination of genetic and epigenetic mechanisms. Thus, we propose to incorporate these parameters into the definition of life.

## 1. Introduction

We can generally recognize and distinguish what is alive from what is not, but it is much more difficult to define in a general way what life is. For the philosopher Heraclitus, a burning flame has a number of analogies with a living system: birth, death, a permanent need for matter, and energy. Like a living being, the flame is a self-sustaining system. In this respect, the concept of autopoiesis transposes this metaphor to living systems, except that it accounts for the permanence of such systems, which can maintain themselves identical while ensuring the replacement of all their components, such as the boat of Theseus in Greek mythology. In any case, there is a long way from the candle flame to the living spark, and a metaphor is not a definition. Aristotle then coined the term “organism” to designate “living beings”. What characterizes such living beings is the complexity of their organization, materialized by the existence of different parts that cooperate with each other. After more than 2000 years of philosophical reflections, one could hope to provide a definitive answer to the question, “What is life?” [1].

On this subject, an anecdote deserves to be told. The great biochemist Daniel Koshland once remembered a symposium bringing together renowned scientists [2] who tried to define life in one sentence [3]. Only one proposal had emerged, with almost unanimous support among the participants: the essence of life is the ability to reproduce itself. Someone then pointed out that according to this definition, a rabbit is not alive [2]: “then one rabbit is dead. Two rabbits—a male and a female—are alive, but either one alone is dead.” Defining life is not an easy task. Moreover, it is always awkward to define something by its potentiality (the capacity to reproduce itself) rather than by its property. If you try to define energy, you will run into the same difficulty [4].

For NASA, life is a “self-sustaining chemical system capable of Darwinian evolution” [5]. Once again, this is a potentiality that is otherwise very vague: “capable of a Darwinian evolution”. In fact, it seems that we need three characteristics to acceptably define a living being, as discussed by Michel Morange [6]: (i) a set of complex molecules, isolated from the external environment by a selective barrier; (ii) the transformation of matter coming from the external environment—we speak of metabolism, a term whose etymology precisely means transformation; (iii) inaccurate reproduction. Why inaccurate? To allow variations and therefore ensure the adaptation and evolution of the system according to external conditions. As a matter of fact, these three characteristics are closely linked. If you remove one leg from a stool, it falls over. Complex molecular structures are necessary to render possible metabolic reactions (enzymes) and also give them the ability to reproduce (nucleic acids). Metabolism is responsible for the synthesis of catalysts (enzymes, ribozymes), which in turn catalyze the chemical reactions of metabolism, which take place under very unfavorable conditions of temperature (37 °C) and pressure (1 atm). Moreover, James Watson and Francis Crick were aware that the double helix structure of DNA suggests an obvious mechanism for the duplication of genetic material, as stated in their seminal publication [7].

Yet the system is not perfect, and this is how it must be. Inaccurate reproduction allows an efficient adaptation of metabolism and molecular structures to environmental changes. (Caution: do not confuse efficient with ideal, because all the possible combinations—therefore infinite—are never tested by nature—whose time, although generous, is limited). In conclusion, these three properties of life (tentatively referred to as the tripod of the living by analogy to Bichat’s physiological tripod of life [8]) constitute the object of biochemistry and define its field of investigation: structural biochemistry for the study of biomolecules, metabolic biochemistry for the study of the chemical reactions in living cells and organisms, and molecular biology for the study of the molecular mechanisms ensuring the (slightly) inexact reproduction of the system.

## 2. Living Systems Are Controlled by Fundamental Parameters

Although these characteristics are obviously useful to describe a living organism, they are not entirely satisfactory because it is impossible to mention the concept of reproduction without coming up against the rabbit paradox mentioned above. In fact, any attempt to define the living based on “the ability to reproduce”, or to show itself “capable of Darwinian evolution” is doomed to failure because of this paradox. We must therefore give up describing the living in terms of reproduction and evolution. Instead, we propose to raise a series of issues that we consider highly specific to life in order to define it, with special emphasis on fundamental parameters that apply iteratively to its different levels of organization, from the subatomic to the cellular and tissular scales. From our point of view, these parameters are time, water, entropy, space, quantum mechanisms, and electrostatic surface potential. These six parameters are, in fact, very linked and most often work together.

### 2.1. Time

Many questions in biology should not be addressed simply by “yes” or “no”, but instead by “it depends on time.” Take the example of hexose sugar, glucose—can this six-carbon molecule freely cross a biological membrane? We should surmise the answer to be “No”, as this sugar is highly polar and is therefore hydrophilic; conversely, a biological semi-permeable membrane is composed of an apolar interior core and is therefore hydrophobic. But in fact, the correct answer is: yes, as this sugar can (as with most other small molecules and ions) cross a membrane, but extremely slowly [9] and therefore this passage cannot take place within the necessary time constraints of a biological system. Studies with membrane bilayers reconstituted with egg or human red cell phosphatidylcholine have established that the upper limit of the glucose permeability coefficient is 10^−10^ cm s^−1^ [10,11]. This value is 6 log_10_ lower than that of the human red blood cell, and this was considered in the late sixties as proof that glucose transport in vivo requires membrane components other than lipids. A second example, perhaps even more significant in biology, concerns enzymes. These catalysts do not make thermodynamically impossible reactions possible but speed up thermodynamically possible reactions to meet the time constraints of biological systems [12]. This has a powerful effect. It has been calculated that the spontaneous rates of biological reactions (i.e., without enzymes) span a range of more than 16 orders of magnitude, with half-times ranging from a few seconds to 1.1 billion years (e.g., for the decarboxylation of amino acids) [13].

A third example is given by signal transduction pathways, traditionally represented as a summary diagram indicating the sequence of events from the initial ligand-receptor interaction at the plasma membrane level to the ultimate effects on gene transcription [14]. Such diagrams are misleading because they compile a series of photographs put end to end to describe the entire process. A video would be much more explicit in understanding how a signal transduction cascade actually works, and most importantly, how long does it take for the signal to be transmitted?

The lifespan of cells in our body may also vary from weeks to years, depending on the tissue. The intestinal epithelium turns over in less than a week, the skin epidermis in a week to a month, and after burning of the tongue, taste buds return in about 2 weeks. Red blood cells have a lifespan of 4 months, which explains why donating 0.5 L of our 5 L of blood every few months does not deplete them. A striking difference in lifespan exists between sperm cells (≈50 days) and oocytes (≈50 years). Fat cells and the skeleton replace themselves in about 10 years, while most of the neurons in the central nervous system and our eye lens cells are not replaced at all throughout our lives. Therefore, taking into account the temporal dimension of typical biological processes, including DNA replication, mRNA transcription, and protein biosynthesis (Table 1), is essential, but we too often forget it [15].

### 2.2. Water

No biological mechanism can be properly explained without analyzing the contribution of water molecules. Indeed, water is the most abundant compound in living organisms [17]. Representative examples include solvent water, water bound to biomolecules, water and hydrogen bonds, entropy, and synaptic transmission [18]. Indeed, water controls the bioavailability of neurotransmitters and thus their diffusion rate in the synaptic cleft, which is a critical aspect of synaptic function [19].

Overall, the most emblematic property of water in living organisms is solubility. A compound is soluble in water if it can form hydrogen bonds with water molecules such that the volume it occupies in the solvent is delimited by a sphere of water molecules. These bound water molecules are constantly exchanged with the water molecules of the bulk solvent. But at any time, the compound is solubilized by an almost constant number of water molecules. The secret of water lies in the very short half-life of hydrogen bonds (1 ps) [20], which allows soluble organic molecules to instantaneously break the network of water molecules in the liquid and compensate for the loss of hydrogen bonds linking water molecules together [21,22]. Thus, the solvent properties of water are strongly dependent on the temporal dimension of water–water and water–compound interactions.

Perhaps more surprisingly, water molecules also control the fidelity of DNA replication through an ingenious control of nucleobase dehydration [23]. The replacement of the 2′OH group of ribose in RNA by 2′H in DNA after the emergence of life on this planet is a solution found by nature to the intrinsic instability of RNA molecules in water. Indeed, the 3′-5′ phosphodiester bond within an RNA molecule is highly thermodynamically prone to hydrolysis by water—hence, DNA is better suited for storing biological information compared to RNA [24].

### 2.3. Entropy

Another essential property of water in biology is its entropic contribution. It is perhaps one of the most neglected fundamental parameters discussed in this review. Statistical mechanics gives a concrete idea of entropy. It involves the notion of irreversibility, which then corresponds to an evolution of the arrangement of molecules towards the most probable arrangement. It is precisely this most probable arrangement that corresponds to the maximum entropy because it allows the total energy of a system to be distributed in the greatest number of possible and distinguishable ways [25]. Technically, entropy cannot be assimilated to the disorder of a system of molecules but to the quantification of this disorder [25,26]. In living organisms, entropy defines the course of any molecular development. It is the key parameter that directs the thread of evolution and determines its irreversibility by indicating the direction of time [27]. The internal entropy of a living being is maintained at low levels at the cost of a high level of energy consumption. In fact, we can consider that a living organism memorizes information, which results in a high degree of organization and therefore a reduction in entropy [27]. This reduction in internal entropy is accompanied by a strong increase in the entropy of the global environment, which includes the water molecules whose disorganization accompanies this structuring. Overall, entropy increases so that living matter obeys the laws of thermodynamics. This contribution of water molecules, through their propensity to create disorder while living matter is structured, is at the origin of a large number of mechanistic possibilities for molecular interactions.

**Figure 1:**
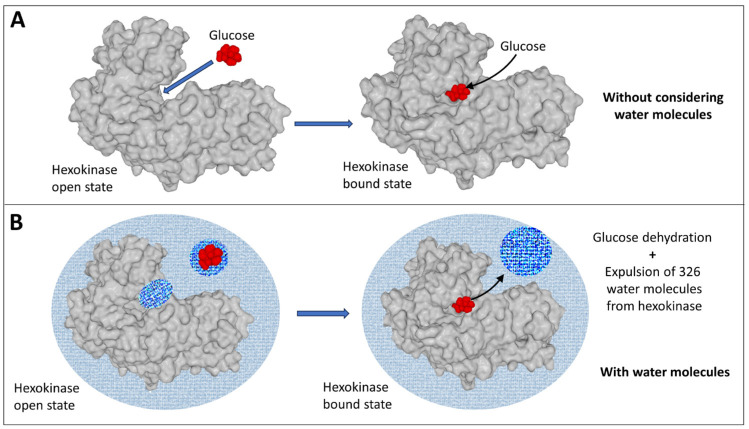
To form the hexokinase/glucose complex, the two molecules must first dehydrate. The departure of water molecules from hexokinase induces a conformational change in the enzyme, which unmasks the active site of the enzyme. The driving force behind this mechanism is the increase in entropy caused by the departure of water molecules during the dehydration step. If we represent the formation of the glucose-hexokinase complex without taking water molecules into account (**A**), we reduce this mechanism to two partners, while water plays a major role (**B**). This picture is a free interpretation of the data published by Reid and Rand [29].

Hexokinase, which catalyzes the phosphorylation of glucose by ATP, provides a striking example of the impact of entropy in biology. The catalytic action of hexokinase consists first of fixing and optimally orienting its two substrates, glucose and ATP. Crystallographic data have demonstrated that hexokinase first binds glucose, which induces a conformational change, multiplying by 50 the affinity of the enzyme for ATP [28]. The formation of the ternary complex seems to defy the laws of thermodynamics because it seems to increase order in the system. The driving force of this process comes chiefly from the movement of 326 water molecules, which leave the enzyme and create disorder through an increase of entropy (Figure 1). The order-disorder balance of the reaction is therefore very clearly in favor of disorder: three molecules order themselves (hexokinase, glucose, and ATP), while more than 300 molecules become disordered. Entropy, which is a quantification of molecular disorder [25], therefore increases during the process.

### 2.4. Space

The spatial dimension is also a mechanism for accelerating chemical reactions. A reduced space concentrates biomolecules, which facilitates intermolecular contacts [30]. In kinetics, time and space (in terms of molecular confinement) are equivalent influences. An important aspect of the impact of space in biology is the reduction in dimensionality, which occurs when a molecule moves from a 3D space to a 2D surface such as a biological membrane [31].

Our group has applied this concept to explain how a virus is attracted by a ganglioside-enriched lipid raft on the plasma membrane of a host cell [32,33,34] and how amyloid proteins form oligomeric channels in neuronal membranes [35,36]. This mechanism, which is at the origin of the concentrating effect of biological membranes, requires fine tuning. The attraction for the membrane must be strong enough to be effective but weak enough to allow lateral diffusion of the compound bound to the membrane, whether physiological or pathological. This explains why it generally involves poorly specific electrostatic forces that govern these interactions, with lipid rafts being globally electronegative and ligands bearing electropositive domains.

### 2.5. Quantum Mechanisms

**Figure 2:**
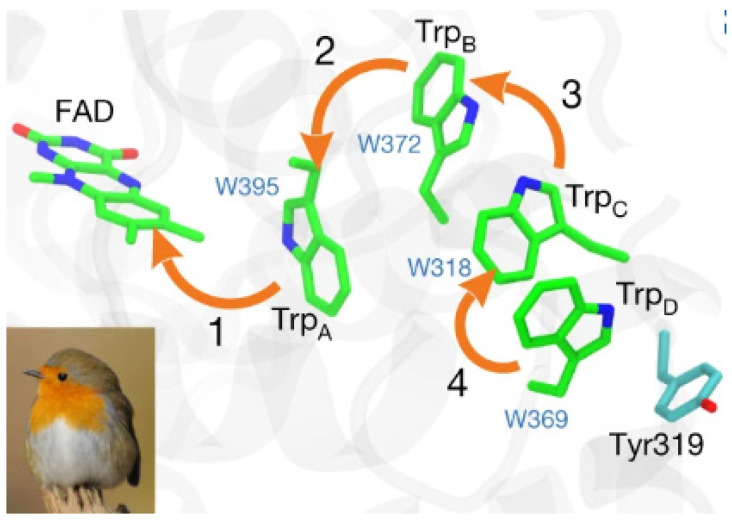
Electron transfers inside the CRY4 protein (orange arrows). The orange arrows indicate the four sequential electron transfers. Only the isoalloxazine part of the FAD is shown. From Wong et al. [49] (published by the Royal Society under the terms of the Creative Commons Attribution License, http://creativecommons.org/licenses/by/4.0/ (accessed on 31 January 2024), which permits unrestricted use, provided the original author and source are credited).

What do photosynthesis, bird migration, and mutations in DNA have in common? Quantum mechanics [37,38]. The contributions of this discipline to biology are such that a new field of research has recently opened up in biology, quantum biology [39]. Quantum mechanisms make it possible to explain fundamental biological mechanisms for which we had significant gaps, such as migratory bird magnetoreception [40], photosynthesis [37], enzymatic catalysis [41], mutations [42], odorant recognition [43], anesthetic effects [44] and perhaps even consciousness [45]. It is understandable that most biologists may be put off by quantum biology, the foundations of which are based on the difficult equations and sometimes obscure interpretations of quantum mechanics [46]. It will therefore not be a question of juggling with such complex equations but of adopting a practical and intuitive strategy by analyzing the impact of quantum phenomena on specific examples such as DNA mutations (tunnel effect) [42] or the swipe card model of signal transduction [43]. Stacking interactions involving aromatic rings with π-bond delocalization [47] will also be considered in this review. Yet there is one scientific field for which quantum biology has given a new potential and striking solution to an old enigma, and that is bird migration. An initial theory proposed that migratory birds have a retina with the particularity of containing a molecule composed of a pair of entangled electrons with zero total spin [48]. By being exposed to light photons, this molecule would cause the decoherence (end of quantum entanglement linked to macroscopic disturbances in the environment) of these two electrons, and the bird would then become sensitive to the earth’s magnetic field. Indeed, laboratory experiments showed that tilting the magnetic field affects the two electrons differently and disrupts this entangled system. This imbalance then causes a chemical reaction, which is transmitted to the bird’s brain by a nerve impulse. In other words, the birds are equipped with a macroscopic device (a “quantum compass”), capable of detecting the Earth’s magnetic field, thus allowing birds to distinguish north from south during their annual migrations [40]. A protein that could serve as a quantum magnetic sensor has been identified in bird eyes: CRY4 (cryptochrome 4). Experimental results suggested that CRY4′s magnetic sensing capabilities are initiated when blue light hits the protein. This light triggers a series of electron transfers that propagate inside the protein through a chain of aromatic residues (Figure 2).

### 2.6. Electrostatic Surface Potential

The crucial importance of this electrostatic property requires special and thorough attention to fully understand biological mechanisms. It is essential to highlight the role of electrostatic potential through concrete examples such as the mode of infection and the evolution of enveloped viruses [33,50,51,52,53,54]. The electrostatic potential determines the distribution of electric charges not only on the surface of biomolecules but also on biological membranes. Its main characteristic is that it defines specific positively or negatively charged areas. The charge distribution gradients can then be visualized by molecular modeling through color variations ranging from blue (positive areas) to red (negative areas), with the electrostatically neutral areas being represented in white according to a universal color code [55].

## 3. From Hydrocyanic Acid to the Double Helix

### 3.1. From HCN to Adenine

**Figure 3:**
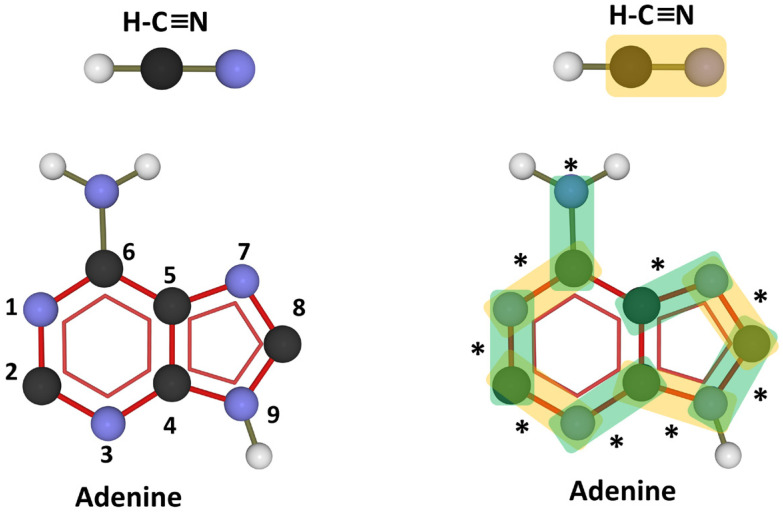
From hydrocyanic acid (H-C≡N) to adenine. Carbon atoms are colored black, nitrogen in blue, and hydrogen in grey. The aromatic base atoms are numbered 1 through 9. An excellent exercise given to students is to highlight the traces of hydrocyanic acid in the adenine molecule (C-N units identified by an asterisk) to understand the prebiotic origin of the base and where its nitrogen atoms come from. For clarity, some C-N units are highlighted in orange, the others in green.

The prebiotic chemistry of adenine (either on earth or in space) is relatively simple since the molecule is synthesized by polymerization of a single precursor, hydrocyanic acid H-C≡N [56]. The energy required for this polymerization reaction is provided by ultraviolet radiation. It is therefore a relatively simple chain polymerization reaction that, with light, leads to adenine. The typical C-N units from hydrocyanic acid are thus easily identifiable in the adenine molecule (Figure 3).

In fact, if the bases present in nucleic acids contain nitrogen, it is because they are derived from hydrocyanic acid. The simplicity of the chemical process for obtaining adenine from hydrocyanic acid suggests that adenine is the first “nucleic” base that appeared on the primitive Earth. This also helps to understand why adenine is overrepresented: in addition to nucleic acids (RNA, DNA), it is also present in ATP (a universal, storable, and usable form of biological energy). The other bases are more difficult to obtain because they have, in addition to the C-N units, oxygen atoms that hydrocyanic acid cannot provide.

### 3.2. Stacking Forces, Attraction of Aromatic Cycles

**Figure 4:**
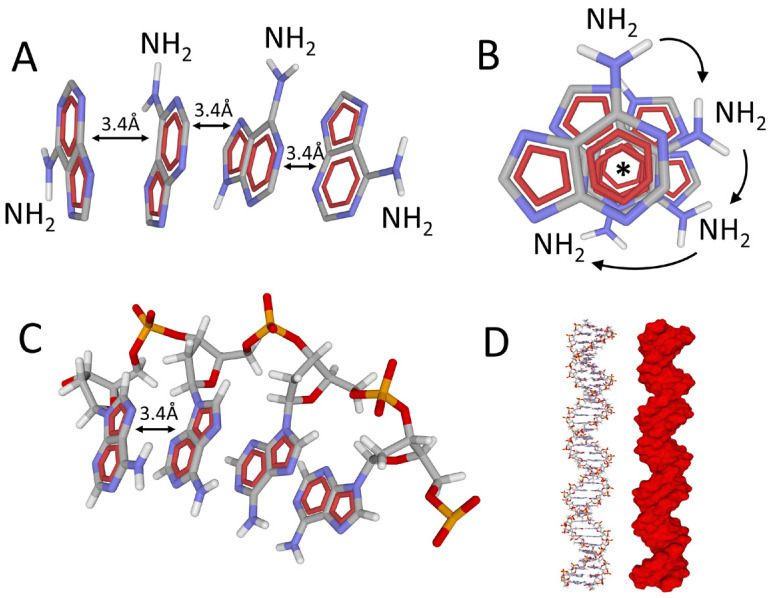
From adenine to DNA. (**A**) self-aggregation of adenine molecules in water. The aromatic cycles stack at 3.4 Å whereas the polar amino group induces a shift between each vicinal adenine molecule (**B**). Note the regular alignment of the aromatic 6-atom ring (asterisk) and the successive shifts of the NH_2_ groups (arrows). (**C**) This typical attraction/repulsion organization is also found in DNA, with the same 3.4 Å space between two adenine units. (**D**) It is remarkable that this very simple mechanism is the basis of the sophisticated regular structure of the DNA double helix (represented in atomic sticks and in surface electrostatic potential, the red color indicating the electronegative zones due to the anionic peripheral phosphate groups).

The aromatic structure of adenine severely limits its solubility in water [57]. When two molecules of adenine meet, they overlap by making their respective aromatic rings coincide. The attraction of aromatic rings leads to relatively well-structured adenine stacks. These formations are stabilized by stacking forces acting between two aromatic rings (Figure 4). The Anglo-Saxon term “stacking” designates two simultaneous properties: stacking and packing. When two aromatic cycles overlap, their π-electron clouds synchronize, creating attractive partial charges δ^+^ and δ^−^ (as in the case of London dispersion forces). These are therefore non-covalent interactions of the van der Waals type [58]. Stacking forces are found in proteins (they participate in their 3D structure), in DNA (stabilization of the double helix), and they also control the interaction of proteins with sugars (through CH-π interactions) [57,59].

Aromatic stacking is a quantum phenomenon that is at the origin of the aggregation of adenine in water. Because of its aromatic cycles, adenine is a relatively apolar molecule, which therefore has a low solubility in water [57]. However, adenine stacks do not occur in a disordered or stochastic fashion. There is a structural feature that determines a characteristic geometry: the presence of the amino (–NH_2_) group. This chemical group shifts each adenine molecule by a constant angle so that the structure gradually adopts a helical shape (Figure 4A,B). In water, adenine molecules stack together in self-organized aggregates, forming a short helix that strongly resembles a strand of the DNA double helix (Figure 4C): distance between two successive adenine rings = 3.4 Å [60]. In this respect, one could consider that the double helix structure of DNA (Figure 4D) is “pre-coded” in the adenine molecule. However, the direction of the helix is exclusively determined by the stereochemistry of the deoxyribose, which is the only constituent with asymmetric carbon atoms.

### 3.3. RNA, Colossus with Feet of Clay

RNA is considered to be the first type of nucleic acid to appear on earth (“RNA world”). The RNA molecule is a polymer of ribonucleotides. Consider that initially, the first ribonucleotides are derived from adenosine. To evolve from adenosine to ribonucleotides, a phosphate group is needed. This does not raise any difficulty since the phosphate group is in fact itself a derivative of the phosphoric acid H_3_PO_4_ whose origin is mineral, therefore abiotic. It is the phosphate group that confers the “acidic” properties of nucleic acids, but at pH 7, this phosphate group is normally deprotonated, so nucleic acids are in fact—in biology—polyanions. Accordingly, their surface potential is globally electronegative, as illustrated for the DNA double helix (Figure 4D), and this is the reason why histones, the principal proteins of chromatin, are cationic [61,62].

Why was RNA not sufficient to allow the development of life? As explained in biochemistry textbooks [63], the answer is simple: RNA is not stable in water because of a weak point. This weak point is its 2′OH group (carried by ribose), which is in the immediate vicinity of the phosphorus atom of the phospho-di-ester bond. When the 2′-OH group is attacked by an OH^−^ ion (generated by water), it releases a proton, and the resulting 2′O^−^ anion breaks the phosphodiester bond. Cut in two, the RNA chain loses its biological significance. In other words, the biological information contained in RNA is at the mercy of a single OH^−^ ion.

Since biological information cannot be stored in an unstable molecule, the RNA needed to be “improved”. This improvement consisted of eliminating the root cause of the problem, i.e., the 2′OH group. By replacing this group with a hydrogen atom, the problem was definitively solved, and thus DNA was invented. The transition from ribose to 2-deoxyribose makes it possible to obtain a water-stable nucleic acid, at least at the level of its 3′-5′ phospho-di-ester bonds. DNA was then used to store biological information in a permanent way. RNA, because of its self-destructive properties, was confined to roles that are certainly important (vehicles of biological information for mRNA, protein translation for tRNA, regulations for microRNA) but limited in time. Biology is being put in place gradually. The temporal dimension takes on its full meaning in the establishment and operation of biological systems.

But you can’t have it all. By losing its 2′OH group and adopting the double helix structure, DNA gives up a property that RNA has retained until today: catalysis. Catalytic RNAs are called ribozymes, the most emblematic being ribosomes, ribonucleoproteins whose catalytic power is entirely held by one of the RNAs that constitute them [64]. However, the catalysis of RNAs is limited to solvent exposed nucleobases and to the 2′OH group [65]. The emergence of proteins has made it possible, thanks to enzymes, to diversify the catalytic possibilities of biomolecules. Interestingly, a means of protecting RNA from water is to surround it with a solid protein capsid itself wrapped in a lipid envelope. This is the basic structure of RNA viruses [66]. In this way, the lifespan of infectious viruses with an RNA genome can reach several days [67] and some RNA viruses can even survive in the sea [68]. Incidentally, their existence is proof of the possible early role of RNA as the essential storage for genetic information.

### 3.4. DNA

**Figure 5:**
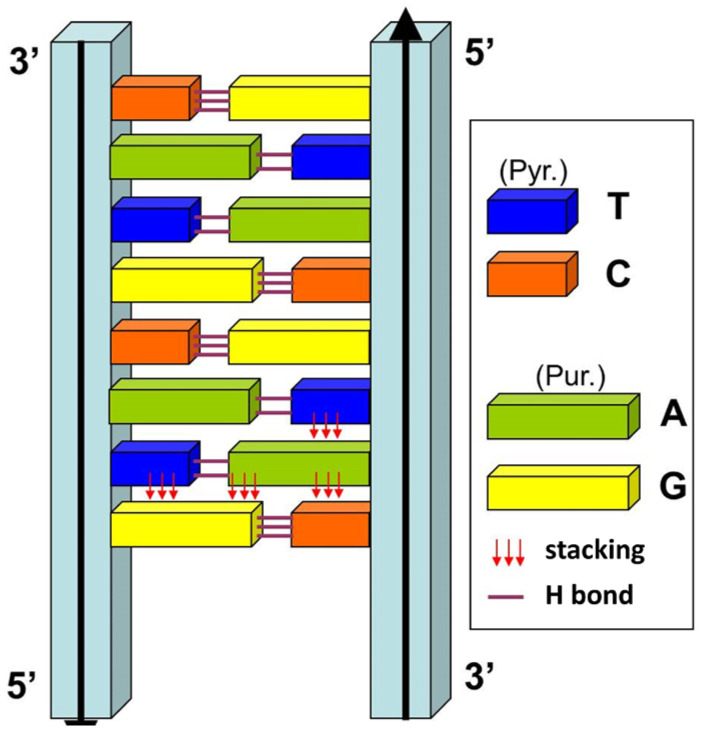
How the DNA double helix is stabilized. This simplified model of the double helix highlights the intra- and inter-strand stacking forces (red arrows), which mainly contribute to the stability of the DNA double helix. The base size dissymmetry (large size for purine bases, small size for pyrimidine bases) and the Pur-Pyr pairing mode (large-small) explain why the stacking forces apply between the two strands.

Everyone “knows” the double helix structure of DNA: two antiparallel chains (therefore oriented, with the notion of 5′-3′ and 3′-5′ polarity) formed by a sugar-phosphate backbone (2-deoxyribose 3′-5′ phosphate), on which are grafted in a perpendicular plane nitrogenous bases (A, T, G, C) interacting by H-bonds. This model is based on the complementarity of the bases, which are paired according to an ultra-classical mode, the “canonical” mode of James Watson and Francis Crick [7]. In this model, it clearly appears that the bases interact according to two complementary mechanisms: by the superposition of their aromatic nuclei (π-π stacking) and by hydrogen bonds (H bonds). The stacking is operative between two successive bases of the same strand, but also between the strands, due to the existence of a large and a small size for the bases (purines = large size, pyrimidines = small size) (Figure 5). Contrary to the classical conception perpetuated by many biologists, H bonds contribute only in a minor way to the stability of the double helix, which instead is stabilized by π-π stacking, a typical quantum mechanism [69,70,71].

## 4. Analogies between DNA and Membranes: Key Role of Surface Potential

### 4.1. Surface Potential of DNA

How is this structure stabilized while allowing the separation of the two strands? At physiological pH, the phosphodiester bonds uniting the nucleotides are negatively charged (PO_4_^−^ phosphate anions). Thus, DNA displays a highly electronegative surface potential [61] (Figure 4D). Moreover, it is surrounded by multiple layers of water. This hydration is necessary for the stability of the double helix: the layers of water shield the negative charges of DNA and reduce the repulsions between phosphate groups, which otherwise would cause the denaturation of the DNA double helix by separation of the strands. This stabilizing role is reinforced in biological environments by the presence of dissolved positive countercharges (mainly Na^+^ ions). This physicochemical property implies that the proteins that bind to DNA (DNA-binding proteins) must be basic (positively charged at physiological pH). As discussed above, this is the case of histones in eukaryotes. In this respect, epigenetic mechanisms can be interpreted as a logical consequence of the electronegative potential of DNA, complemented by electropositive proteins that control how biological information is delivered through subtle changes in their surface potential, which impact gene expression. In other words, the histone code must be electrostatic [72].

### 4.2. Surface Potential of Biological Membranes: Lipid Rafts

**Figure 6:**
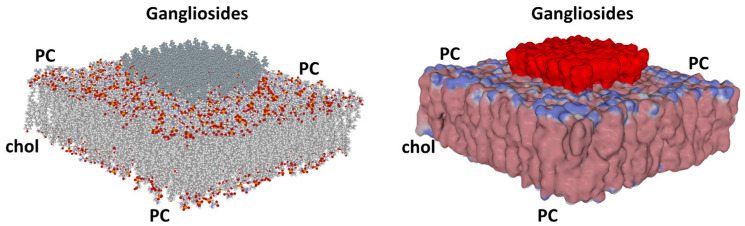
What a lipid raft looks like from a molecular and an electrostatic point of view. Gangliosides are colored in grey (left panel), oxygen atoms in red, phosphorus in yellow, and hydrocarbon chains in two-tone grey. A cholesterol molecule (chol) is visible on the left edge of the membrane. The electrostatic surface potential is represented on the right panel. surface Electronegative areas are colored red, electropositive areas are blue, and neutral areas are white/pale pink. Note that the raft emerges from the plasma membrane bilayer higher than bulk lipids (zwitterionic phosphatidylcholine displays both blue and red zones). PC, phosphatidylcholine. The gangliosides protrude at the membrane interface, giving lipid rafts an optimal situation that facilitates the attraction of biomolecules and pathogens. From Matveeva et al. [34] (published by MDPI under the terms and conditions of the Creative Commons Attribution (CC BY) license (https://creativecommons.org/licenses/by/4.0/ (accessed on 31 January 2024), which permits unrestricted use, provided the original author and source are credited).

The fluid mosaic membrane model [73] has been useful in understanding how proteins fit into a lipid bilayer. It has thus made it possible to define proteins possessing one or more transmembrane (TM) domains and to define their topology. However, this model proved insufficient to account for the biochemical diversity of lipids and heterogeneity in cholesterol distribution. In 1977, Simons and Ikonen published a seminal paper that redefined the membrane into a mosaic of domains called lipid rafts [74]. These rafts are condensed domains of the plasma membrane enriched in cholesterol and sphingolipids. Among these sphingolipids, the glycosphingolipids confer on these domains a negative electrostatic potential, the extent of which is modulated by the content of gangliosides, which are anionic glycosphingolipids [50,75]. Thus, an interesting correlation can be established between the electronegative surface of a lipid raft [76] (Figure 6), and the DNA double helix [77] (Figure 4D).

There is a striking analogy between the epigenetic mechanisms controlled by histone–DNA and protein–raft interactions. Although this concept is well-established for histones and DNA, whose interactions determine different levels of gene expression/repression (histone code) [72], it was only recently that lipid rafts were recognized to exert epigenetic-like mechanisms on membrane proteins [78]. This new concept developed by our team is based on the demonstration that lipid rafts act as conformational platforms for membrane proteins and control their function through a second level of epigenetic mechanisms. We called this raft-dependent epigenetic function the “epigenetic dimension of protein structure”, a concept referring to any non-coded conformational information that applies to a protein beyond the genetic code [78,79].

Overall, lipid rafts play a critical role in cell biology and, due to their electrostatic properties, are privileged sites of invasion by viruses, bacteria, parasites, and prions that display complementary electropositive surfaces [50]. The co-evolution of these pathogens with host cell membranes is a critical factor explaining the gradual occurrence of virus variants and quasi-species, as demonstrated for HIV-1 and SARS-CoV-2 [33].

## 5. How DNA Polymerases Control the Fidelity of Replication

### 5.1. Geometry of the DNA Binding Pocket

A DNA polymerase is an enzyme that synthesizes a complementary strand of DNA by conforming to the instructions (sequence of bases) of a strand of DNA or RNA called ‘template’. A distinction is therefore made between DNA-dependent and RNA-dependent DNA polymerases, depending on the nature of the template strand to be copied. There are several different DNA polymerases in eubacteria and eukaryotes with distinct structures, but all of them work globally with the same basic principles [80].

Roughly, a DNA polymerase is shaped like a right hand, with the thumb, fingers, and palm forming three distinct domains. It has two types of substrates: the strand to be copied (the template strand) paired with the primer (the primer strand) and the nucleoside 5′-triphosphate (dNTP to be incorporated). Let’s leave aside the problem of the primer. What is particularly striking compared to other enzymes is the quality of these substrates, which are in fact generic, and not specified. Indeed, the chemical nature of these substrates varies in nucleotide sequence for the template strand to be copied and its complementary primer. Moreover, there are four distinct possible dNTPs (dATP, dTTP, dGTP, and dCTP) that can be incorporated into the newly synthesized DNA chain. The active site of the enzyme must deal with this particularity. The dNTP-binding pocket must therefore be able to accommodate the four types of nucleotides.

The fidelity of incorporation is, of course, a fundamental criterion of the activity of DNA polymerases. For a long time, the ability to form H-bonds between complementary bases was thought to be responsible for correct pairing [81]. This hypothesis has not received experimental confirmation. Indeed, the DNA polymerase is able to continue the polymerization even when a base of the template strand is missing (an abasic site); it preferentially incorporates dATP, which is the base presenting the more important stacking activity [23]. In addition, the enzyme can make a strand of DNA by incorporating modified (fluorinated) nucleotides unable to form H-bonds, or even a polycyclic aromatic compound as exotic for biology as a dNTP analogue whose base has been replaced by pyrene. In this case, the major criterion has been clearly identified as aromatic stacking, a quantum mechanism.

H-bonds are therefore not responsible for pairing specificity during DNA replication. In fact, the dNTP-binding pocket is made in such a way that the space available for the dNTP to incorporate depends on the nucleotide facing it on the strand to be copied: in front of Adenine, there is only room for Thymine but not for Cytosine [82]. It is a geometric selection, and H-bonds do not play any role in this selection. The same mechanism operates for all base pairings, which are strongly dependent on steric constraints imposed by the architecture of the complex between the enzyme and its substrates and not on the ability of the selected bases to form H-bonds.

### 5.2. How Water Controls Fidelity

Finally, to illustrate once again the fundamental role of water in biology, note that the two bases that will pair up must first dehydrate. The energy lost during this dehydration step will be recovered by the formation of H-bonds between the bases. Incidentally, this is also the main role of these H-bonds (and not the stabilization of the double helix, which is mainly ensured by stacking forces). The intervention of water molecules in the process of DNA replication explains why pairings between two small bases (e.g., T-T) are strongly disadvantaged, although the size of the active site of the enzyme does not exclude it. In this case, the two hydrated T occupy a larger space than the pocket of the enzyme, and they do not dehydrate because the energy lost cannot be compensated by the formation of the H bond between the two T [23].

## 6. Mutations

### 6.1. Hydrogen Tunneling: A Logical Consequence of the Double Helix Structure

In 1963, Löwdin proposed a proton tunneling model to explain the occurrence of spontaneous point mutations in DNA base pairs. According to this concept, each proton in a hydrogen bond linking A⋅T or G⋅C base pairs can be transferred by quantum mechanical tunneling (or quantum jumping) from the position corresponding to the normal base pair to a non-natural tautomeric structure (denoted with *) [42,83]. In this case, A* will no longer combine with T but with C, and several other combinations will be obtained: A-C*, G*-T, and G-T* [42]. Overall, the movement of a single proton through an H bond associating with a base pair of the double helix will destroy the genetic message. It cannot escape anyone that this mechanism would not occur if DNA were not a complementary double helix resulting from strict base pairing rules. In other words, it is indeed the double helix structure that carries with it this propensity to mutations, which means that the reproduction of living organisms is never perfectly exact. Hence the evolution. Stop the tunnel effect; you will annihilate the spontaneous occurrence of mutations. Ming-Feng Lu et al. observed that the replacement of hydrogen by deuterium (bacterial culture in D_2_O instead of H_2_O) significantly decreased the mutational rate of Bacillus cereus [84]. Since deuterium atoms cannot travel through H bonds via quantum jumping, these data were interpreted as a preliminary experimental confirmation of the Löwdin DNA mutation model. It thus appears that one of the fundamental characteristics of life is its ability to program itself its evolutionary potential through quantum mechanisms. Mutations are then no longer considered the consequences of flaws in the cell replication apparatus but as variations that are certainly random yet definitely programmed. Recent data on the evolution of a minicell indicated that mutation rates are not affected by genome minimization [85], which can be viewed as an indirect argument supporting the notion that mutations are an integral part of the program of life, independently of genome size.

### 6.2. Mutations Creating a Stop Codon or inducing a Change of Amino Acid

Mutations in genomic DNA (or RNA, for some viruses) are expressed at the protein level. If we consider the typical case of a point mutation, we have to distinguish three possibilities: (i) the creation of a stop codon; (ii) the change of a base of the codon involves a change of amino acid; and (iii) the change of a base of the codon does not modify the amino acid because the genetic code has the particularity of being degenerate (the same amino acid can be coded by several codons). The effect of a stop codon results in the interruption of translation and consequently the production of a truncated protein (Table 2), which no longer has its biological activity or possibly has another.

According to the possible effects listed in Table 2, an amino acid change can lead to modifications in: (i) the electrostatic potential (e.g., cation → neutral, aromatic → neutral); (ii) the solvation by water molecules (e.g., cation → neutral, aromatic → cation); and (iii) the folding of the protein due to chemical incompatibility or steric clash.

However, a clear distinction should be made between conservative substitutions that correspond to the replacement of one amino acid by another with similar physicochemical properties (e.g., Asp to Glu, Trp to Phe, or Arg to Lys) and non-conservative changes when the wild-type and the mutated amino acid belong to distinct classes (e.g., Phe to Glu or Ser to Trp) [86].

### 6.3. When Time Encoded in DNA Is Transferred into Conformational Information at the Protein Level: The Intriguing Case of Silent Mutations

Consider now the case of silent mutations. Many biologists would probably intuitively consider that these mutations have no influence on the 3D structure of the protein since they do not cause amino acid changes. Indeed, this is often the case, but not always [87,88]. In fact, when the same amino acid is encoded by several different codons, the cell must manufacture and make available the corresponding tRNAs to the ribosome [89]. For instance, in the case of glycine, four tRNAs are required, but their intracellular concentration is not equivalent [90]. Therefore, these tRNAs cannot be mobilized in the same way, which will affect the speed of translation. Thus, a tRNA present in high concentration will allow rapid protein biosynthesis, while a lower concentrated tRNA will cause a slowdown in the speed of translation at the level of the amino acid coded by the mutated codon [91]. Time is thus an essential control element in the acquisition of the 3D structure of the protein because the amino acids must interact with selected partners to build a functional protein [92].

A representative example concerns a glycine codon located immediately after a lysine codon. In this case, lysine is incorporated in the peptide chain just before glycine. Lysine has a flexible hydrocarbon chain with 4 CH_2_ groups terminated by an NH_3_^+^ cation. This configuration can generate a large degree of conformational freedom, allowing the terminal cations group to test several orientations before it finds its specific partner, which can be either a negative charge (Asp or Glu), an oxygen atom from a peptidic bond, or a hydroxylated amino acid (Ser, Thr, or Tyr). Delaying the incorporation of the next residue (Gly) will allow Lys to test more partners than initially programmed by the genetic coding, potentially leading to incorrect conformations. Interestingly, a single silent substitution in the genome of apple stem grooving virus (ASGV) has been shown to attenuate virus-infection symptoms [93]. The detection of many silent mutations in the genome of SARS-CoV-2 [94] variants should encourage researchers to assess the impact of these mutations on the structure of viral proteins, and therefore on their function. Recently, silent mutations have been associated with significant changes (most often reductions) in yeast fitness [95]. Another study demonstrated that silent mutations can alter enzyme structure and function through a kinetic effect [96], giving a supplemental demonstration of the tRNA selection model [91,92].

## 7. Structure or Not Structure: A Protein Dilemma Solved by Fundamental Parameters

The possibility of controlling the speed of translation through intracellular concentrations of tRNA gives living beings intriguing diversity possibilities that could not exist if the genetic code were not redundant. Thus, it is possible that the maintenance of such a code after billions of years of evolution responds to a necessity on which the pressure of selection can be exerted. The fundamental parameter that controls this level of regulation is time. By modulating the speed of translation via the relative frequencies of tRNA levels, time acts directly on the acquisition of the 3D structure of proteins. This mechanism, of course, applies to proteins that have a sTable 3D structure. However, one of the great advances gained by deciphering the human genome was the discovery that not all proteins have a 3D structure. In fact, the classic model for the acquisition of the 3D structure of proteins is based on the hydrophobic effect, which applies to apolar amino acids. In an aqueous medium, these group together at the center of the protein structure so as to minimize thermodynamically unfavorable interactions with water molecules. This process, referred to as hydrophobic collapse, warrants that the structuring of a protein takes place spontaneously in a very short period of time (≈60 ns at 305 K) [97]. Concomitantly, the polar amino acids are rejected at the periphery of the structure, where they establish H-bonds with surrounding water molecules. The ability of proteins to fold so quickly despite almost theoretically infinite possibilities of conformations (due to a very large number of degrees of freedom in an unfolded polypeptide chain) is known as Levinthal’s paradox [98]. Although the hydrophobic collapse model solves another temporal paradox of biology, it nevertheless presents an intrinsic weakness for proteins that do not display a harmonious distribution balance between apolar and polar amino acids.

What happens when the protein has an excess of polar amino acids? The protein is then perfectly at ease in the middle of water molecules, with which it establishes a large number of H-bonds without, however, adopting a particular folding. It oscillates between thousands of serpentine conformations, all perfectly compatible with water. These proteins are called IDPs (intrinsically disordered proteins) [99,100,101,102,103]. The great conformational flexibility of IDPs allows them to interact with multiple ligands, depending on their location and temporality (cellular and nychthemeral cycles). Up to 50% of the proteins encoded by the human genome are IDPS or have an IDP domain [104]. These proteins are involved in critical biological functions such as cell division, differentiation, and signal transduction. The fundamental parameters that control the biological activity of IDPs are water (which determines their conformational flexibility) and time (which determines the availability of ligands during the life cycles of cells, tissues, or organisms).

In a sense, IDPs can be interpreted as a typical example of programmed chaos. The program is encoded by the genetic code; the chaos is a watery affair. Because they do not adopt a stable structure, IDPs are a major limitation of artificial intelligence programs such as AlphaFold [105]. Despite triumphant media claims that AlphaFold has solved the problem of protein structure, it is obvious that IDPs are, by essence, not concerned by this revolution [106]. Membrane proteins, which are confronted with various lipid environments, are another remarkable exception to the field of application of AlphaFold [78]. Lipid chaperones complement genetic information by modulating the conformation of membrane proteins and acting as functional switches by selecting appropriate conformations according to the cell’s needs at a specific time [78,79].

We are far from able to model how living organisms handle programmed chaos through environmental fluctuations, e.g., lipid raft association or dissociation. However, some recent progress is worth mentioning. By using molecular modeling approaches, we showed that an initial interaction of an initially disordered extracellular loop of a membrane protein triggers a conformational wave that reaches the intracellular domain and affects its conformation. In the lipid raft, the conformational wave is propagated through cholesterol in close interaction with a TM domain. This approach could be used for other receptors whose activity is lipid raft-dependent. The fundamental parameters that control transmembrane waves are time (the whole process is speeded up by the co-localization of gangliosides and cholesterol), water (the initial disorder of the extracellular loop), quantum mechanisms (movements of electrons, CH-π stacking of ganglioside–protein interactions, swipe card model of signal transduction), and electrostatic potential (electronegative field of lipid rafts, electropositivity of receptors).

## 8. The Fundamental Roots of Life

From a chemical point of view, all known living organisms, from bacteria to humans, are made up of the same basic components that can be referred to as fundamental bricks: nucleobases, amino acids, sugars, and fatty acids, all made of carbon, hydrogen, oxygen, nitrogen, and other elements (sulfur, phosphorus) in smaller quantities. In this respect, terrestrial life can be assimilated to a molecular lego based on organic chemistry. The interstellar medium contains lots of organic molecules, which are called by astronomers iCOMs (for interstellar Complex Organic Molecules) [107]. The Nobel laureate Christian De Duve hypothesized that the chemical seeds of life are universal [108]. According to Vladimir Uversky’s group, the recent discovery of mechanisms for the synthesis of simple oligopeptides under conditions relevant to the astrophysical environment raises hopes in the search for extraterrestrial life [109]. If these assumptions are correct, then it is possible that life could emerge elsewhere in the universe and not only on Earth.

**Figure 7:**
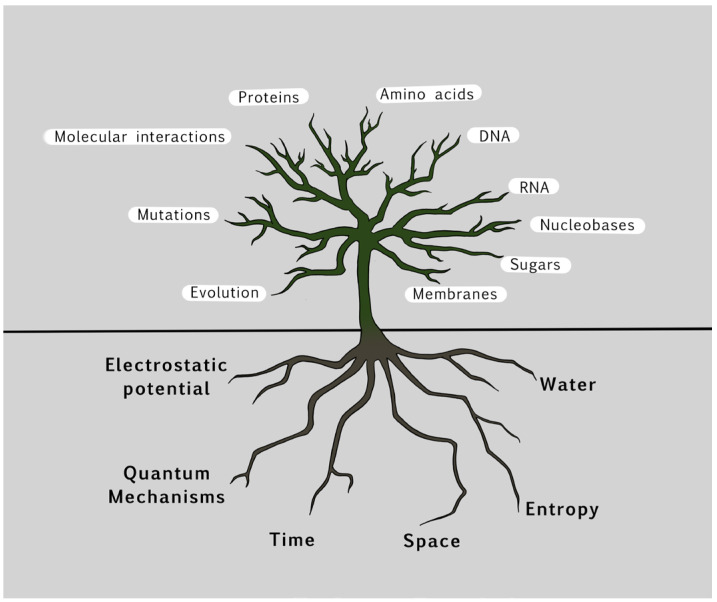
Highlighting the roots of biology. The fundamental parameters are represented as root networks that feed the tree with universal rules. The combined action of these parameters allowed the emergence of life on our planet, and potentially elsewhere in the universe.

De Duve’s intuition is almost a scientific fact. The conservation of the dimensions of atomic clouds, and therefore the size of atoms and the resulting chemical properties, is based on the foundations of quantum physics. There is only one way to construct the atoms and therefore the periodic table of the elements from the Schrödinger equation and the Pauli exclusion principle, and the solution will therefore always be the same in any planetary system through the universe, which implies the universal identity of matter. Now, if we mix the fundamental parameters considered in this review with the universal seeds of life, we can propose a new kind of tree representation, distinct from classic phylogenetic trees. We do not contest that phylogenetic trees have had great success in biology because they allow an easy visualization of the relationships between different living organisms [110,111]. Our approach is different (Figure 7).

Our representation considers the fundamental parameters as the roots of biology. These parameters propagate through the trunk to create critical biological entities and characteristic properties of life based on fundamental organic bricks. The explosion of life manifests itself in the branches of the tree, which do not correspond to the classic [archae-prokarya-eukarya] Tree of Life but to representative and highly critical examples of biomolecules controlling biological mechanisms and entities, all constrained by the fundamental parameters. The path of life can take many possible directions; these fundamental parameters will always apply. In other words, if life disappeared and then reappeared later on Earth or elsewhere in the universe, the classic Tree of Life would become obsolete because the new paths taken by the new organisms would most likely be different from the previous ones. However, the roots of biology, based on the universal application of fundamental parameters to any living organism, would remain valid.

## 9. Predictable Effects of Fundamental Parameters

A summary of predictable effects based on fundamental parameters, focusing on the examples treated in the present review, is given in Table 3.

As a matter of fact, these parameters most often occur in a combined manner. For instance, the electrostatic surface potential of viruses determines their kinetics of infection and their evolution, as shown for SARS-CoV-2 variants [50] and HIV-1 quasi-species [33]. Water and entropy are generally acting together. The arrangement of adenine aggregates in water [60] (that prefigures the double helix geometry) results from the influence of water, entropy, electrostatic potential, and quantum mechanisms. Table 3 shows that the fundamental parameters studied in this review make it possible to explain numerous biological mechanisms but also to predict some, for example, the evolution of viruses.

However, not everything can be predicted by these parameters. Infections, in particular, can modulate certain species and lead them down a completely unpredictable path. This is the case of the deletion of a critical 92-base pair exon in one ancestral hominin CMAH gene, almost two million years ago, just before the emergence of the genus *Homo* [128]. The consequence is that the human species, instead of expressing two types of surface sialic acids, Neu5Gc and Neu5Ac, like all mammals, has only Neu5Ac. Ajit Varki has suggested a possible selection mechanism involving a Neu5Gc-binding pathogen responsible for malaria [128]. The implications of this gene inactivation have been felt until today, one of the most worrying being the consumption of meat enriched in Neu5Gc. This sialic acid is recycled into our own glycoconjugates, a condition that can trigger an autoimmune disease [129]. In a typical Red Queen model [130], the Plasmodium falciparum malaria parasite has then evolved to use human Neu5Ac as a binding determinant for invading our red blood cells [131]. This evolution could be perfectly predicted by the fundamental parameters, but not by the initial event. This illustrates why classic phylogenetic trees are so unique.

## 10. Conclusions and Perspectives

In this article, we seek to identify what is explanatory in biology. It is in this sense that we highlight six fundamental parameters (the list is not exhaustive) that may explain universal biological mechanisms, which constitute the very essence of biology as it emerges from the tripod concept of living beings. Reproduction, in its Janus-like double face, is perhaps the most emblematic example: the fidelity of DNA replication controlled by water, mutations generated by quantum mechanisms (tunnel effect). Time then appears to be the ultimate controller of biology, exerting its effects on the frequency of biological mechanisms, underlying all its manifestations. It is here that our concept meets Carlo Rovelli’s view on the nature of time [15], whose sub-layers and intricate networks constitute the major roots of the tree representation of biology proposed in this article. Finally, the electrostatic potential should be considered as one of the drivers of the evolution of pathogenic agents, regulating by Maxwell’s laws the temporal dimension of host–pathogen interactions at the molecular level. Space and entropy, which control many biological processes, also belong to these fundamental parameters.

The tripod of the living that we described in the introduction of this review is an attempt to condense the main characteristics of the living into a plural definition. Our approach is different. We propose to consider time, space, water, entropy, quantum mechanisms, and electrostatic potential as fundamental parameters that control the emergence and expansion of life on this planet. This angle of view allows us to reinterpret the origin of living organisms, considering basic parameters that are often neglected in biology textbooks. We believe that these parameters are critical for better understanding what life is, and how it handles order and chaos through a combination of genetic and epigenetic mechanisms. They should not be considered vital principles because they obey the fundamental laws of physics and chemistry. They also do not explain, by themselves, the origin of life on earth. Yet we can consider that life, if it emerges, is the consequence of the quantum properties of atoms, which are identical everywhere in the universe and onto which fundamental parameters apply, themselves matching these quantum properties.

## Figures and Tables

**Table 1:** Characteristic rates and timescales in cell biology (from ref Shamir et al. [16]).

	Cell Cycle	DNA Replication	Transcription	Translation	mRNA Half-Life	Cell Movement	Diffusion over 1 µm
**Bacteria** **(*E. coli*)**	1 h	10^3^ nt/min	10–100 nt/s 1 min/gene [1 kbp]	10 aa/s 1 min/protein [300 aa]	10 min	10 µm/s	10–100 ms
**Eukarya** **(HeLa cells)**	1 day	10^3^ nt/min	10–100 nt/s 10 min/gene [10 kbp]	10 aa/s 1 min/protein [300 aa]	10 hr	1 µm/s	1–10 s

**Table 2:** Examples of possible effects of non-silent mutations.

Initial Codon	Mutation	Consequence
**UAU**	**UAA**	STOP → truncated protein
**GAU**	**GAA**	Asp → Glu (anion → anion)
**CGU**	**GGU**	Arg → Gly (cation → neutral)
**UUU**	**UAU**	Phe → Tyr (aromatic → aromatic)
**UGG**	**AGG**	Trp → Arg (aromatic → cation)

**Table 3:** Example of predictable effects of fundamental parameters in living organisms.

Fundamental Parameter	Examples of Predictable Effects	References
**Time**	✓ Requirement of catalysis ✓ Mechanism of action of enzymes and ribozymes ✓ Membrane transporters ✓ Half-life of hydrogen bonds in water ✓ Effect of silent mutations on protein folding	[13] [112,113] [114] [20] [87,92]
**Water**	✓ Solubility of biomolecules ✓ Synaptic transmission ✓ Control of DNA replication fidelity ✓ Intrinsically disordered proteins ✓ Intrinsic limitations of AlphaFold ✓ Entropy-driven mechanisms	[57] [18,19] [82] [100,101] [78,79] [115,116]
**Entropy**	✓ Organization of biological membranes ✓ Conformational changes ✓ Enzyme catalysis ✓ Molecular interactions	[117,118] [29] [119] [120]
**Space**	✓ Control of enzymatic reactions ✓ Control of molecular interactions ✓ Reduction in dimensionality ✓ Biological membranes as concentrators ✓ Molecular crowding	[121] [30] [31] [18,34] [122]
**Electrostatic potential**	✓ Lipid rafts as signal transduction platforms ✓ Host–pathogen interactions ✓ Virus evolution ✓ Control of neurotransmitter action ✓ Epigenetic regulations	[75] [33] [32,34] [19,123] [72,124]
**Quantum mechanisms**	✓ Nucleobase stacking and DNA double helix ✓ Mutations controled by tunnel effect ✓ Swipe card model of signal transduction ✓ Photosynthesis ✓ Bird migration ✓ Amyloidogenesis	[60] [42] [125] [126] [40] [127]

## Data Availability

Not applicable.
